# Seven genome sequences of bacterial environmental isolates from the western coast of the Greenland Ice Sheet

**DOI:** 10.1128/mra.01192-25

**Published:** 2026-02-23

**Authors:** Markus Dieser, Heidi J. Smith, Kathryn Caruso, Christine M. Foreman

**Affiliations:** 1 Center for Biofilm Engineering, Montana State University 827241, Bozeman, Montana, USA; 2 Department of Chemical & Biological Engineering, Montana State University 33052, Bozeman, Montana, USA; 3 Department of Microbiology and Cell Biology, Montana State University 123776, Bozeman, Montana, USA; University of Wisconsin-Madison, Madison, Wisconsin, USA

**Keywords:** Greenland, Ice Sheet, bacteria, cryosphere

## Abstract

We report the genomic sequences of seven bacterial isolates from the western coast of the Greenland Ice Sheet. The surface ice along the margins of the ice sheet is dominated by microbes. As the ice sheet melts, it is important to understand the ecological role and biotechnological potential of this novel extremophile microbiome.

## ANNOUNCEMENT

Understanding the genomic potential of bacteria from extreme environments, such as the Greenland Ice Sheet, drives the discovery of novel compounds for biotechnological or bioremediation purposes. To aid this discovery, supraglacial meltwater samples were collected in June 2011 in the south of Jakobshavn Isbrae (69.589301N, −49.783762W) in conjunction with a larger sampling effort (1). Bacteria were isolated aerobically on R2A agar plates at 4°C in the dark. Isolates were selected and cultured based on colony morphology. Visual cues, such as colony size, color, shape, elevation, margin, and texture, were used for subculturing. Isolates stored in 25% glycerol at –80°C were grown on R2A agar plates at 4°C for 21 days. Single colonies were inoculated in R2A broth (20 mL) at 4°C while shaking at 150 rpm to late exponential phase. A standard cetyltrimethylammonium bromide (CTAB) procedure was used for extracting DNA (2).

Unless otherwise stated, default settings were used. Standard quality shotgun libraries for bacterial strains GrIS 1.19, GrIS 2.15, GrIS 1.11, GrIS 2.11, GrIS 1.2, and GrIS 2.14 were sequenced on the Illumina NovaSeq S4 platform (2 × 151 bp paired-end reads) (3). Libraries were prepared on the PerkinElmer Sciclone NGS robotic liquid handling workstation using the KAPA Biosystems HyperPrep Library Preparation Kit (Roche). DNA was sheared to 459 bp using a Covaris LE220 focused ultrasonicator. DNA fragments were size selected by a two-step solid-phase reversible immobilization (SPRI) using magnetic beads (Agencourt). Fragments were end-repaired, A-tailed, and ligated with Illumina-compatible sequencing adaptors (IDT). Raw Illumina sequences were quality filtered using BBTools v38.87 (4) and assembled with SPAdes (version v3.15.3; –phred-offset 33 –cov-cutoff auto -t 16 -m 64 –careful -k 25,55,95) (5). Contigs with a length <1 kb (BBTools reformat.sh: minlength=1000 ow=t) were discarded.

For improved high-quality draft genomes, libraries were prepared using the SMRTbell Express Template Prep Kit 2.0 (Pacbio) for the bacterial strain GrIS_2_6 and sequenced on the Pacific Biosciences (PacBio) Sequel II platform (6). DNA was sheared around 10 kb using Megaruptor 3 (Diagenode). Sheared DNA was treated with exonuclease, DNA repair enzyme mix, and end-repair/A-tailing mix and ligated with barcoded overhang adapters using SMRTbell Express Template Prep Kit 2.0 (PacBio). Libraries were purified with AMPure PB Beads (PacBio) and bound to Sequel II polymerase 2.0 using the Sequel II Binding Kit 2.0 (PacBio). Libraries were sequenced using TBD sample-dependent sequencing primers, 8M v1 SMRT cells, and Version 2.0 sequencing chemistry with 1 × 900 sequencing movie run times. Collapsed adapters were removed with BBTools v38.87. Reads >5 kb were assembled with Hierarchical Genome Assembly Process (HGAP) (smrtlink/8.0.0.80529, HGAP 4 [1.0]) using default settings (7). Contigs with a probability of being a plasmid were identified using TensorFlow (8).

CRISPR elements were identified using the programs CRISPR Recognition Tool (CRT) v.1.8.2 (9) or PILER-CR (10). All genomes were annotated in the Integrated Microbial Genomes (IMG) database (≥IMG Annotation Pipeline v.5.0.23) (11). Genome completeness was estimated using checkM v.1.2.2 (12). The genome sequence-acquired 16S ribosomal RNA gene was queried in command line against the SILVA database 138.1 with blastn, from BLAST + 2.13.0 for taxonomic classification (13). Sequence details are provided in Table 1.

**TABLE 1:** Summary of genome characteristics^*a*^^,^^*b*^

Organism [16S rRNA gene % match] accession no.	# Raw reads	Coverage (×)	Completeness (%)	Contamination (%)	# Contigs	L50/N50 (bp)	Size (bp)	GC (%)	# Genes	# C	# P
*Actimicrobium* sp. GrIS 1.19 [98.2] JAXCHT000000V000 SRR25764258	13,196,650	376.6	98.96	0.4	91	11/118,791	4,177,960	60.57	4,008	1	3
*Cryobacterium* sp. GrIS_2_6 [99.6] JAXCHP000000000 SRR25764241	291,115	221.7	98.74	2.15	2	1/4,227,007	4,344,596	66.70	4,361	–	1
*Glaciihabitans* sp. GrIS 2.15 [97.2] JAXCHU000000000 SRR25764244	9,819,950	395.3	99.49	0.51	37	5/279,211	3,782,709	63.50	3,805	1	–
Oxalobacteraceae bacterium sp. GrIS 1.11 [98.9] JBEONR000000000 SRR28961171	13,987,370	276.8	99.55	1.66	178	18/106,551	6,495,031	61.20	6,018	–	–
Oxalobacteraceae bacterium sp. GrIS 2.11 [97.2] JBEONN000000000 SRR28961169	12,670,602	343.7	98.93	1.89	134	11/126,143	4,803,472	51.76	4,560	3	–
*Undibacterium* sp. GrIS 1.2 [98.6] JBEONP000000000 SRR28961168	11,708,220	316.5	99.92	0.3	155	21/79,900	5,120,017	46.89	4,733	2	–
*Variovorax* sp. GrIS 2.14 [99.0] JBJXIA000000000 SRR25764256	19,293,144	271.1	100.0	0.69	99	7/272,887	5,649,109	63.33	5,650	–	2

^*a*^ –, none.

^*b*^ C, CRISPR; P, plasmid.

## Data Availability

The JGI genome project (ID: 505037) is available from the IMG database. The genome sequences have been submitted to GenBank and are available through the accession numbers listed in Table 1.
